# Ehrlenmeyer’s Method

**Published:** 1892-04

**Authors:** 


### ERLENMEYER'S METHOD.

The rapid withdrawal of the drug in the treatment of the morphine habit, as recommended by Erlenmeyer,* and practiced by some physicians, is inhuman,—it is barbarous. Moreover, it is attended with great danger of heart failure, and death has resulted in consequence of it. The sudden and complete, or even very rapid, deprivation, entails a suffering indescribable; it is a crying of the whole nervous system for its accustomed stimulant; every organ is in sympathy, and suffers.

In the *Medical Age*, for February, 1892, Dr. E. P. Hurd reports a case in which he succeeded in breaking the habit, but the woman suffered the tortures of death a thousand fold; few systems could have stood the suffering, and very few could have persisted to the end.

We quote from Dr. Hurd's report of the case. [Third day]:

"Dec. 3d. Patient was so utterly demoralized this morning,

*Treatment of Morphine Habit, Erlenmeyer, in Leisure Library Series, Geo. S. Davis, Publisher.

moaning and beseeching, and at times even screaming, that I gave her three grains of morphine by mouth, which, in half an hour, brought elysium to her. The vomiting and diarrhœa had been troublesome; sulphonal, whiskey, nourishment, had all been vomited.

"Dec. 4th. Mrs. B. was in such a dilapidated condition that I gave her, by mouth, two grains sulphate morphia. The diarrhœa and vomiting still continued, but with lessened frequency and severity.

"Dec. 6th. The morphine was reduced to half a grain, by mouth, with the understanding (to which the patient consented) *that this should be the last dose which should be given.* The allowance of whiskey was increased to one ounce every two hours, if needed. As the previous night had been nearly sleepless, in spite of the sulphonal, it was agreed that chloral should be given the coming night, in suitable doses, to cause sleep.

"Evening visit. Found Mrs. B. nearly frantic; it was 'an indefinable distress all over.' She clung to my hand, and begged most beseechingly for a little morphine. I felt compelled pitilessly to refuse. 'Are *you* going to conquer,' I asked, 'or the *demon?*' I urged her to summon all her will-power, *or she would be lost.* Twenty grains of chloral, with one drachm of ammoniated tincture of valerian, and $\frac{1}{100}$ grain hyoscine hydrobromate, were given, and the nurse was directed to repeat the dose without the hyoscine in two hours if the patient was not relieved.

"Dec. 7th. Patient had slept much of the time the previous night, but this morning was almost ungovernable; was, however, too weak to make any effectual resistance; did little but lament, and beg for stimulants; had consumed a pint of whiskey the last twenty-four hours. Some very marked hysterical manifestations. When I arrived she again implored for morphine, and again I demanded whether *she* were going to conquer or the *demon.* This was the critical time for which we had been looking; if she passed this crisis triumphantly, she would begin to recover her old vigor and energy, and rapidly to recuperate."

It will be observed that a *pint of whiskey* was given the woman in twenty-four hours, and yet the doctor remarks the occurrence of "severe hysterical symptoms;" and then, that the poor, abused and revolting stomach should be called upon (later) to receive four ounces of infusion of valerian,—a most disgusting dose,—is a statement that fills the reader with surprise, and with sympathy for the sufferer. She recovered in spite of the treatment.

The JOURNAL raises its voice in earnest protest against a practice so barbarous, and sincerely hopes that its readers will not be tempted to resort to it. That the habit can be overcome by less painful methods is well established. The drug should be reduced gradually, and the symptoms of nervous perturbation met with appropriate remedies; the digestion should be carefully fostered, for nourishment is of the highest value. A pint of whiskey and infusion of valerian,—to say nothing of the chloral, sulphonal and bromide,—are agents not much calculated to promote digestion. Dr. Hurd should be ashamed to make public the details of his case; it is not at all creditable to him, and we hope no one else will resort to his methods.

